# *SaHsfA4c* From *Sedum alfredii* Hance Enhances Cadmium Tolerance by Regulating ROS-Scavenger Activities and Heat Shock Proteins Expression

**DOI:** 10.3389/fpls.2020.00142

**Published:** 2020-02-28

**Authors:** Shuangshuang Chen, Miao Yu, He Li, Ying Wang, Zhuchou Lu, Yunxing Zhang, Mingying Liu, Guirong Qiao, Longhua Wu, Xiaojiao Han, Renying Zhuo

**Affiliations:** ^1^ State Key Laboratory of Tree Genetics and Breeding, Chinese Academy of Forestry, Beijing, China; ^2^ Key Laboratory of Tree Breeding of Zhejiang Province, The Research Institute of Subtropical Forestry, Chinese Academy of Forestry, Hangzhou, China; ^3^ Institute of Leisure Agriculture, Jiangsu Academy of Agricultural Sciences, Nanjing, China; ^4^ National Engineering Laboratory of Soil Pollution Control and Remediation Technologies, Institute of Soil Science, Chinese Academy of Sciences, Nanjing, China

**Keywords:** *Sedum alfredii* Hance, heat shock transcription factor, cadmium stress, overexpression, ROS-scavenging enzyme

## Abstract

The heat shock transcription factor (Hsf) family, an important member in plant stress response, affects cadmium (Cd) tolerance in plants. In this study, we identified and functionally characterized a transcript of the Hsf A4 subgroup from *Sedum alfredii*. Designated as *SaHsfA4c*, the open reading frame was 1,302 bp long and encoded a putative protein of 433 amino acids containing a complete DNA-binding domain (DBD). Heterologous expression of *SaHsfA4c* in yeast enhanced Cd stress tolerance and accumulation, whereas expression of the alternatively spliced transcript *InSaHsfA4c* which contained an intron and harbored an incomplete DBD, resulted in relatively poor Cd stress tolerance and low Cd accumulation in transgenic yeast. The function of *SaHsfA4c* under Cd stress was characterized in transgenic *Arabidopsis* and non-hyperaccumulation ecotype *S. alfredii*. *SaHsfA4c* was able to rescue the Cd sensitivity of the *Arabidopsis athsfa4c* mutant. *SaHsfA4c* reduced reactive oxygen species (ROS) accumulation and increased the expression of ROS-scavenging enzyme genes and *Hsps* in transgenic lines. The present results suggest that *SaHsfA4c* increases plant resistance to stress by up-regulating the activities of ROS-scavenging enzyme and the expression of *Hsps*.

## Introduction

Heavy metal pollution from industrial and agricultural activities has become a worldwide concern (Akesson et al., 2014). Given that heavy metals are persistent and non-degradable, their contamination of soil is irreversible and leads to a decline in soil fertility as well as in crop yield and quality (Luo et al., 2015). Heavy metals accumulated in plants and animals can enter the food chain and subsequently harm human health (Iqbal et al., 2015). Thus, the remediation of heavy metal-contaminated soil has long been a challenge and a popular research focus. Phytoremediation, in which hyperaccumulator plants remove contaminants from polluted soil, is an eco-friendly and cost-effective remediation measure (Lee, 2013).

Heavy metal hyperaccumulators are species of plants that can naturally accumulate substantial amounts of heavy metals in their aerial organs without showing apparent signs of toxicity (Verbruggen et al., 2013). *Sedum alfredii* Hance (*Crassulaceae*), a species first discovered in mining regions in China (Yang et al., 2002), is a highly valuable material for phytoremediation because of its propensity to hyperaccumulate cadmium (Cd), zinc (Zn) and lead (Pb). Recently, the allied species *Sedum plumbizincicola* was described (Wu et al., 2013). The physiological mechanisms underlying this species’ resistance and hyperaccumulation of different metal ions have been investigated (Lu et al., 2008; Jin et al., 2008; Li et al., 2016; Tian et al., 2016; Tian et al., 2017). Analyses of the transcriptome, small RNAs, degradome and proteome have been carried out under Cd stress (Li et al., 2016; Han et al., 2016; Peng et al., 2017). Furthermore, many genes associated with Cd uptake, resistance and hyperaccumulation, such as *SpHMA3*, *SpMTL*, *SaREF*, *SaMT2*, *SaCAX2* and *SaCu/ZnSOD*, have been characterized from *S. plumbizincicola* and *S. alfredii* (Zhang et al., 2014; Liu et al., 2016; Zhang et al., 2016; Li et al., 2017; Liu et al., 2017; Peng et al., 2017). Despite these findings, transcription factors, which play an important role in abiotic stress, remain largely unknown in *S. alfredii*.

Heat shock transcription factors (*Hsfs*) comprise an important family of transcription factors involved in plant growth, development, signal transduction and stress response. Numerous studies have reported that the *Hsfs* of diverse plant species show different characteristics in response to abiotic stresses. *Hsfs* respond to external heat stress through activating the expression of heat shock proteins (Hsps) and other downstream genes by binding specifically the heat shock element (HSE) in the promoters of *Hsf*-responsive genes (Yamaguchi-Shinozaki and Shinozaki, 2006; Scharf et al., 2012; Wang et al., 2016). *Hsfs* are also involved in heavy metal tolerance. For instance, *HsfA1a* confers Cd tolerance in tomato (*Solanum lycopersicum* L. cv Ailsa Craig) by partially upregulating *Hsps* expression (Cai et al., 2017). In *HsfA1a*-silenced tomato plants, melatonin levels were down-regulated, whereas in *HsfA1a*-expressing plants the melatonin biosynthetic gene caffeic acid *O*-methyltransferase 1 (*COMT1*) was up-regulated and melatonin accumulation was induced under Cd treatment (Cai et al., 2017). The downstream gene metallothionein of *HsfA4a* is critical to enhance Cd tolerance in wheat (*Triticum aestivum*) and rice (*Oryza sativa*) (Shim et al., 2009). These findings demonstrate that *Hsfs* can bind to HSEs in the promoters of heavy metal stress-responsive genes to regulate the expression of downstream genes.

We previously observed that 18 *SaHsfs* showed a high expression level among the 22 members of the *Hsf* gene family identified in hyperaccumulation ecotype (HE) *S. alfredii* after Cd treatment. Furthermore, *SaHsfA4c* improves the Cd tolerance and accumulation in transgenic yeast (Chen et al., 2018). The function of *SaHsfA4c* in the regulation of Cd tolerance, however, remains unknown. In the present study, we analyzed the functions and subcellular location of *SaHsfA4c* from the HE *S. alfredii*. We concluded that overexpression of *SaHsfA4c* in *Arabidopsis* and non-hyperaccumulation ecotype (NHE) *S. alfredii* enhanced Cd tolerance by regulating the activities of reactive oxygen species (ROS)–scavenging enzymes and *Hsps* expression. *SaHsfA4c* is thus a potentially valuable gene for phytoremediation.

## Materials and Methods

### Plant Materials and Growth Conditions

Hyperaccumulation and non-hyperaccumulation ecotype *S. alfredii* were collected from mining regions in Quzhou City, China, and a tea plantation in Hangzhou City, China, respectively. The shoots were excised and cultivated in half-strength Hoagland-Arnon solution (pH 5.8). The nutrient solution was renewed every 3 days. After three weeks, robust and uniform plantlets (approximately 10 cm in height) were selected and cultured in half-strength Hoagland-Arnon nutrient solution containing 400 μM CdCl_2_. Plants were harvested and separated into roots, stems and leaves after 0, 0.5, 1, 6 and 12 h of treatment. The plants were grown in an artificial climate chamber under controlled conditions with a long-day photoperiod (16 h light/8 h dark) at 25°C.

*Arabidopsis thaliana* (Col-0) plants and transgenic *Arabidopsis* were grown under sterile conditions using half-strength Murashige and Skoog medium under the same conditions as *S. alfredii*. Seven-day-old plants were then incubated in Hoagland-Arnon nutrient solution for one month. Root and leaf samples were obtained after treatment with nutrient solution supplemented with or without 30 µM CdCl_2_ for one week. All experiments were performed in triplicate.

### Gene Isolated, Identification and Plasmid Construction

To clone *SaHsfA4c*, total RNA was isolated from HE *S. alfredii* using the Total RNA Purification Kit (Aidlab, Beijing, China). cDNA was synthesized from 1 µg total RNA using the SuperScript^®^ III First-Strand Synthesis System (Invitrogen, Carlsbad, CA, USA) according to the manufacturer’s instructions. The full-length *SaHsfA4c* cDNA was isolated from HE *S. alfredii* using the species-specific primers *SaHsfA4c*-F/R designed based on the transcriptome sequences. In addition, the genomic sequence of *SaHsfA4c* was PCR-amplified from genomic DNA, which was isolated from the leaf of HE *S. alfredii* using the cetyltrimethyl ammonium bromide method. The obtained sequences were confirmed by sequencing and Blast analysis against the NCBI database (http://www.ncbi.nlm.nih.gov/). The Heatster platform (https://applbio.biologie.uni-frankfurt.de/hsf/heatster/) was used for sequence identification and domain analysis.

The purified PCR products were first ligated into the pDONR222 vector (Invitrogen, Carlsbad, USA) using the Gateway^®^ BP Clonase reaction. This construct was then sequenced and recombined with the destination vectors pK7WGF2.0, pYES-DEST52 and pH2GW7.0 using the Gateway^®^ LP Clonase reaction to generate subcellular localization, yeast, and plant expression vectors, respectively.

### Phylogenetic Analysis

Deduced amino acid sequences containing the N-terminal region, DNA binding domain (DBD), and oligomerization domain (HR-A/B regions) of *SaHsfA4c* and other HsfA4 members from different plant species were used to compile a multiple sequence alignment. The sequence alignment was generated using ClustalX software and visualized with GeneDoc. Neighbor-joining trees were constructed using MEGA 6.0 with 1000 bootstrap repetitions performed to evaluate statistical support for the tree topology. Information on the sequences analyzed is given in **Supplementary Table S1**.

### Quantitative Real-Time-PCR, Western Blotting and Immunolocalization

SYBR-based Quantitative real-time PCR (qRT-PCR) mixtures (TaKaRa, Da Lian, China) were prepared according to the manufacturer’s instructions and analyzed on a 7300 Real-Time PCR System (Applied Biosystems, Foster City, CA, USA). At least three biological replicates, with three technical replicates, were performed per sample. *UBC9* and *TUB* served as internal controls for HE and NHE *S. alfredii* (Sang et al., 2013), whereas *AtActin* was used for *Arabidopsis*. Relative expression levels were calculated using the 2^−ΔΔCt^ method. To construct a heat-map, the original expression levels of the analyzed genes were first normalized by the z-score method. And then, the normalized mRNA levels of the wild type (WT) under the non-stress condition (Control) were set arbitrarily to 1. Finally, the results were displayed using HemI software (The CUCKOO Workgroup, Wuhan, China). Values ranging from −2 to +2 were used to indicate the absolute signal intensity, and red and green colors were used to represent high and low expression values, respectively. All primers used in this analysis are listed in **Supplementary Table S2**.

A specific peptide (KEDNIKYDGLLTMH) for the SaHsfA4c protein was synthesized and used to produce polyclonal antibodies in rabbits (Abmart, Shanghai, China). The root, stem, and leaf from HE *S. alfredii* were ground into powder in liquid nitrogen and incubated with extraction buffer (150 mM NaCl, 50 mM Tris-HCl (pH 7.5), 1 mM PMSF, and 0.5% NP-40) at room temperature for 5 min. The lysate was centrifuged at 12,000 rpm for 15 min and the supernatant was retained. The protein concentration was quantified with the Enhanced BCA Protein Assay Kit (Beyotime, Shanghai, China). A total of 30 μg of protein per sample was used for SDS–PAGE and western blotting. Western blotting and immunolocalization were performed in accordance with methods detailed in previous studies (Han et al., 2017).

### Subcellular Localization

The subcellular location of SaHsfA4c protein was determined by polyethylene glycol-mediated transformation of *Arabidopsis* protoplasts according the description by Yoo et al. (2007). The empty vector p35S-GFP was used as control. *Arabidopsis* protoplasts transformed with pK7WGF2.0-SaHsfA4c were observed under a LSM510 confocal laser scanning microscope (Carl Zeiss, Oberkochen, Germany).

### Cadmium Tolerance Analysis in Yeast

The lithium acetate method was used to introduce expression constructs into *Saccharomyces cerevisiae* mutant strain *Δycf1* (Szczypka et al., 1994). The transformants pYES-DEST52-*SaHsfA4c* and pYES-DEST52-*InSaHsfA4c* and yeast containing the empty vector pYES2.0, were cultured in liquid synthetic dextrose−uracil medium until OD_600_ up to the logarithmic phase. The bacteria solutions were then diluted serially (OD_600_ = 10^0^, 10^−1^, 10^−2^, 10^−3^, 10^−4^ and 10^−5^) and spotted on synthetic galactose–uracil agar plates supplemented with CdCl_2_ (0, 15, 30, or 50 µM). Photographs were taken after incubation at 28°C for 3 days. To evaluate the growth status of yeast transformants in SG-U liquid medium containing 15 µM CdCl_2_, the OD_600_ was measured every 12 h for 96 h*. The* Cd content was measured as described by Li et al. (2017). The empty vector pYES2.0 was used as the control in each experiment. At least three biological replicates, with three technical replicates, were performed per analysis.

### Plant Transformation and Physiological Analysis

The floral dip method (Clough and Bent, 1998) and a previously reported protocol (Liu et al., 2017) were applied for transformation of *Arabidopsis* and NHE *S. alfredii*, respectively, using *Agrobacterium tumefaciens* strain EHA105 carrying pH2GW7.0-*SaHsfA4c*. An *Arabidopsis AtHsfA4c* T-DNA insertion line (SALK_086202; *athsfa4c*) was obtained from the SALK collection, and homozygous mutants were used for the phenotype rescue experiment. The following transgenic plant lines with high transcript levels of *SaHsfA4c* were selected for subsequent experiments. *oxSaHsfA4c*#*1* and *oxSaHsfA4c*#*2* for overexpression in *Arabidopsis*, *athsfa4c/SaHsfA4c*#*1* and *athsfa4c/SaHsfA4c*#*2* for rescue of the *Arabidopsis* mutant *athsfa4c*, and *oxSaHsfA4c*#*4′*, *oxSaHsfA4c#7′* and *oxSaHsfA4c#10′* for overexpression in NHE *S. alfredii*.

To investigate the potential effects of *SaHsfA4c* in plants, leaves of *Arabidopsis* were stained with 3,3*′*diaminobenzidine (DAB) and nitroblue tetrazolium (NBT) to reveal *in situ* accumulation of two ROS indices, namely hydrogen peroxide (H_2_O_2_) and superoxide anion (O_2_^−^), respectively. Leaves from the same location in each plant were harvested and immersed in DAB (1 mg∙ml^−1^ DAB in ddH_2_O, pH 3.8) or NBT solution (1 mg∙ml^−1^ NBT in 10 mM phosphate buffer, pH 7.8), respectively. The samples were then incubated at room temperature for 12 h in darkness. Finally, the leaves were bleached by boiling in ethanol for 20 min to remove chlorophyll. Brown and blue spots respectively indicated the presence of H_2_O_2_ and O_2_^−^ *in situ* (Thordal-Christensen et al., 1997; Wohlgemuth et al., 2002). The activities of ROS-scavenging enzymes, including ascorbate peroxidase (APX), catalase (CAT) and peroxidase (POD), were measured. Ice-cold phosphate buffer was added to one gram of frozen, pulverized leaf samples. The homogenate was centrifuged with 12,000 rpm at 4°C for 10 min, and the supernatant was used for enzyme activity determination. The activities of APX, CAT, and POD were measured by absorption photometry as described by Aebi (1984); Ullah et al. (2016) and Polle et al. (1994), respectively. All experiments were repeated three times independently.

### Co-Expression Network Construction

Gene co-expression relationships in HE *S. alfredii* were determined from published transcriptome data under Cd stress (Han et al., 2016). To obtain insights into the functions of gene co-expressed with *SaHsfA4c*, gene ontology (GO) terms were analyzed using osgo (http://www.omicshare.com/tools/Home/Soft/osgo). Finally, *Hsfs*, *Hsps* and antioxidant activity-related genes were selected to construct the co-expression subnetwork of *SaHsfA4c* (**Supplementary Table S3**). The subnetwork was visualized using Cytoscape software (Smoot et al., 2011).

### Statistical Analyses

For a given biological sample, three or more representative technical repeats were analyzed and the mean value for the biological sample was determined. Data were calculated as the means ± standard deviation (SD) of three or more independent biological repeats of each sample. SPSS package 20.0 (IBM Corporation, Armonk, NY, USA) and Origin 8.5 (OriginLab, Northampton, MA, USA) software were used to perform the statistical analyses and display the results, respectively. The significance of differences among multiple groups was evaluated by one-way ANOVA followed by Tukey’s multiple range test (*p* = 0.05).

## Results

### Gene Isolation and Bioinformatic Analysis

**Figure 1:**
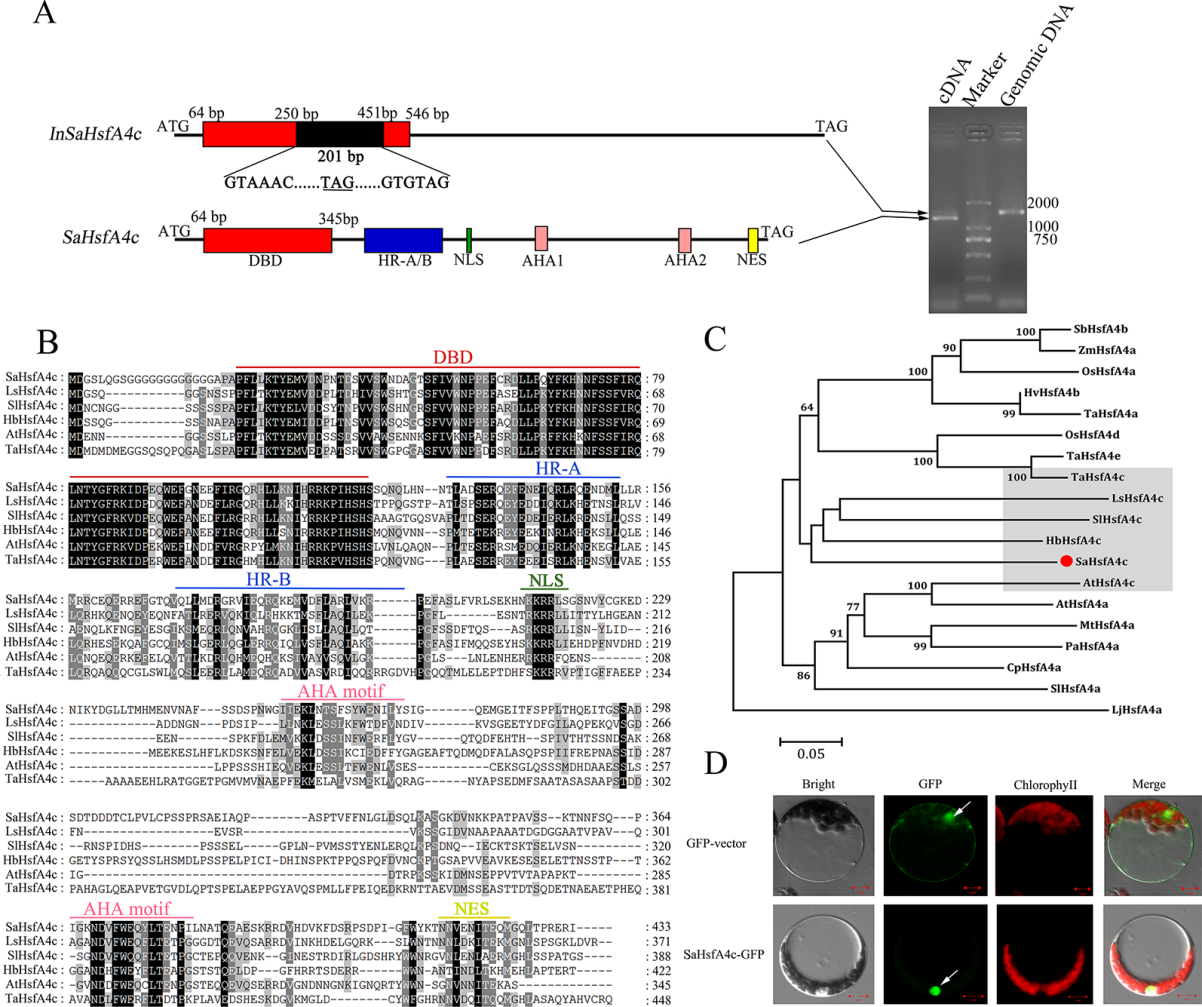
Identification of SaHsfA4c. **(A)** Schematic diagram of the *SaHsfA4c* and its two transcripts (Left). A 201 bp intron existed in the *InSaHsfA4c* mRNA, introducing a new stop codon. The new stop codons are underlined. Genomic DNA or cDNA was used as the template for PCR amplification of *SaHsfA4c* gene (Right). **(B)** Comparing amino acids sequence from SaHsfA4c and its orthologs. **(C)** Phylogenetic analysis of SaHsfA4c and other Hsf proteins. The species of origin of the HsfA4 s are indicated by the abbreviation before the gene names: SbHsfA4b, XP_002456359.1, *Sorghum bicolor*; ZmHsfA4a, CAA58117.1,*Zea mays*; OsHsfA4a, AK109856, *Oryza sativa*; OsHsfA4d, AK100412, *Oryza sativa*; HvHsfA4b, AEB26589.1, *Hordeum vulgare* subsp. Vulgare; TaHsfA4a, ACN93796.2, *Triticum aestivum*; TaHsfA4c, AHZ44767, *Triticum aestivum*; TaHsfA4e, AHZ44768, *Triticum aestivum*; LsHsfA4c, XP_023766107.1, *Lactuca sativa*; SlHsfA4a, XP_010317423.1, *Solanum lycopersicum*; SlHsfA4c, XP_004243245.1, *Solanum lycopersicum*; HbHsfA4c, XP_021689590.1, *Hevea brasiliensis*; AtHsfA4a, NP_193623.1, *Arabidopsis thaliana*; AtHsfA4c, NP_199383.1, *Arabidopsis thaliana*; MtHsfA4a, XP_003629847.1, *Medicago truncatula*; PaHsfA4a, AAL12248.1, *Phaseolus acutifolius*; CpHsfA4a, XP_021890826.1, *Carica papaya*. **(D)** Subcellular localization of SaHsfA4c. White arrows indicate the nucleus of cell. Control, p35S-GFP vector; SaHsfA4c-GFP, SaHsfA4c fused with GFP. Scale bar = 10 μm.

The full-length coding sequence of *SaHsfA4c* was cloned and confirmed by sequencing. Interestingly, two PCR products were detected by gel electrophoresis (**Figure 1A**). DNA sequencing revealed that *SaHsfA4c* was alternatively spliced into two distinct transcripts of 1,302 and 1,503 bp length. The longer transcript, with a 201 bp intron, was present in the *SaHsfA4c* genomic DNA sequence; thus, the two transcripts were designated *SaHsfA4c* and *InSaHsfA4c*. The *SaHsfA4c* transcript encoded 433 amino acids and included a conserved DBD domain, an intermediate HR-A/B region, a nuclear location signal (NLS), two transcriptional activation domains (AHA motifs) and one nuclear export signal (NES) predicted using the Heatster online tool (**Figure 1B**). The second transcript, *InSaHsfA4c*, contains an advanced stop codon; it encoded a truncated protein variant of 109 amino acids, harbored an incomplete DBD, and lacked both the HR-A/B and AHA motifs (**Figure 1A**). Phylogenetic analysis revealed that SaHsfA4c belongs to the HsfA4c subgroup, as it showed a high similarity to other known HsfA4c proteins (**Figure 1C**).

The predicted NLS domain located between the HR-A/B region and the AHA1 motif suggested a putative nuclear targeting of SaHsfA4c, and the SaHsfA4c protein contained NLS sequences (KKRR). To test this hypothesis, transient expression analysis in *Arabidopsis* protoplasts was conducted to verify the subcellular localization of the SaHsfA4c protein. An expression construct containing SaHsfA4c fused with GFP was introduced into the protoplasts. Green fluorescence signals were mainly detected in the nucleus of cells. p35S-GFP, used as the control vector, was expressed throughout the cells (**Figure 1D**).

### Expression Patterns and Immunolocalization

**Figure 2:**
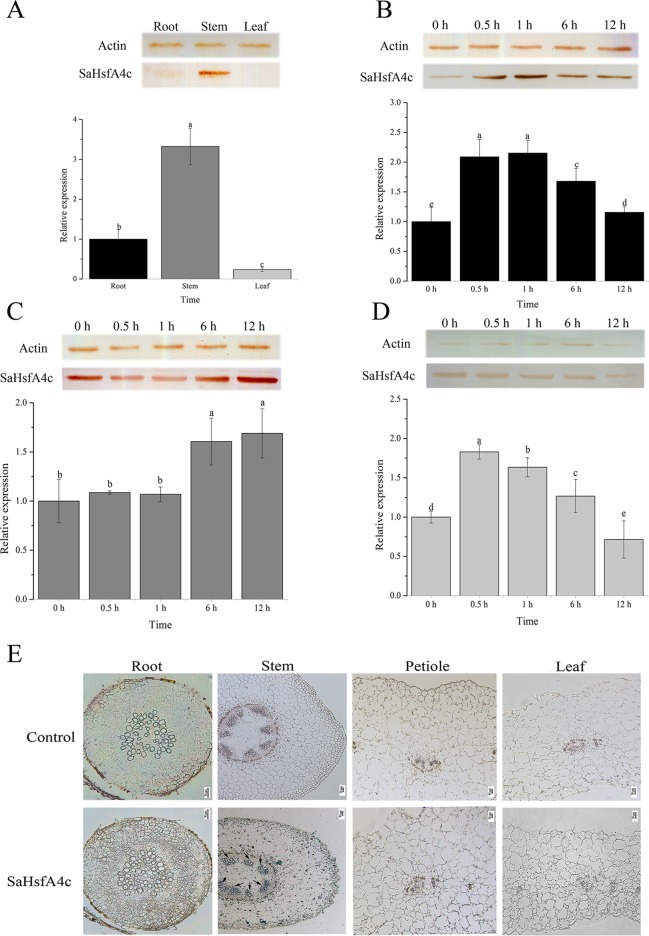
Expression patterns and immunolocalization of SaHsfA4c. **(A–D)** Expression patterns of *SaHsfA4c* under Cd treatment. Western blotting to quantify SaHsfA4c protein accumulation in treated tissues was present as SDS-PAGE gel, and the expression profiles of *SaHsfA4c* in *S. alfredii* treated with CdCl_2_ for different amounts of time were displayed in histogram. The normalized mRNA levels of the root **(A)** and tissues **(B–D)** without treatment were set arbitrarily to 1, respectively. **(A)** Without Cd treatment. **(E)** Immunolocalization of SaHsfA4c. Black arrows indicate the hybridization signals. A total of 30 μg of protein per sample was used for SDS–PAGE and western blotting. Different letters on the bars indicate significant difference among multiple groups according to Tukey’s multiple range test (*p* = 0.05).

On the basis of qRT-PCR analysis, *SaHsfA4c* was expressed under the non-stress condition in all three tissues analyzed (root, stem and leaf) of HE *S. alfredii*. The highest expression level was detected in the stem, followed by the root (**Figure 2A**). Under Cd stress, *SaHsfA4c* transcript and protein levels increased in all tissues. *SaHsfA4c* expression attained a peak of 2.15- and 1.82-fold at 1 h and 0.5 h after Cd stress in the root and leaf, respectively, and then gradually decreased (**Figures 2B, C**). In contrast, *SaHsfA4c* expression in the stem increased gradually, and the *SaHsfA4c* transcript level after 12 h treatment was 1.68-fold higher than that before treatment (**Figure 2D**). Similar trends in SaHsfA4c protein levels with or without exposure to Cd stress were observed.

To determine the tissue specificity of SaHsfA4c, immunolocalization was carried out using root, stem, leaf and petiole sections. Highly specific SaHsfA4c signals were mainly detected in stem xylem cells, with weaker signals observed in the root, petiole, and leaf. No signals were detected in the control (non-hybridization with SaHsfA4c) (**Figure 2E**). These results were generally consistent with the expression patterns of SaHsfA4c obtained by qRT-PCR and western blotting, which suggested that the SaHsfA4c protein specifically localized to the stem in HE *S. alfredii.*

### The DBD of *SaHsfA4c* Is Vital for Cd Tolerance in Yeast

**Figure 3:**
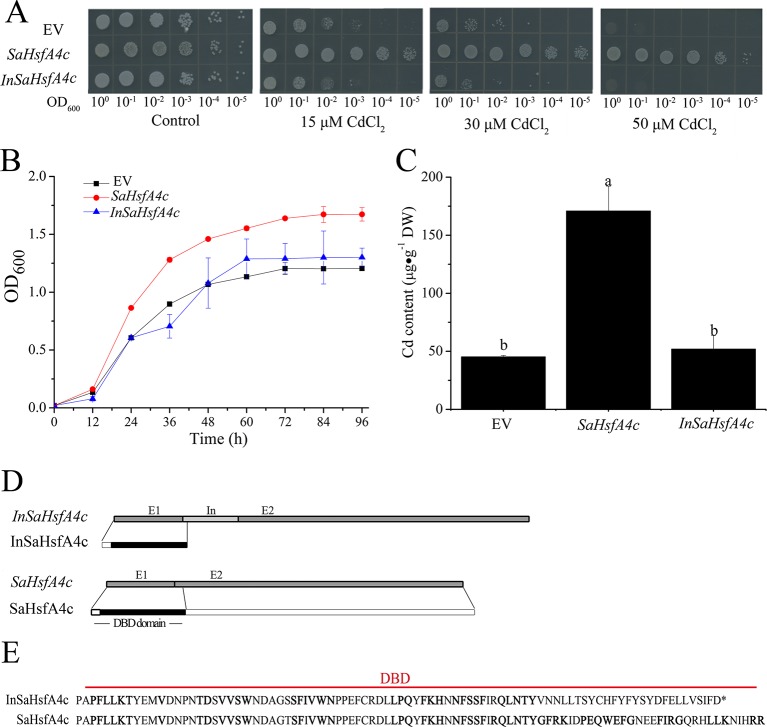
*SaHsfA4c* increased Cd tolerance in transgenic yeast. **(A)** Cd tolerance assay. **(B)** The time course growth of each yeast strains was measured by OD_600_. **(C)** Cd content. **(D)** Transcript and protein structures of SaHsfA4c and InSaHsfA4c. E1, E2 and In represent exon 1, 2 and intron. **(E)** The sequences of DBD in SaHsfA4c and InSaHsfA4c.

To determine their influence on Cd tolerance, *SaHsfA4c* and *InSaHsfA4c* transcripts were recombined in the yeast expression vector pYES-DEST52. Interestingly, only *SaHsfA4c* substantially enhanced Cd tolerance in *Δycf1*. In the absence of CdCl_2_, no significant difference in colony number or size was observed between the empty vector (EV) and *Δycf1* harboring *SaHsfA4c* or *InSaHsfA4c*. On SG-U medium supplemented with 15, 30, or 50 μM CdCl_2_, the growth of the three transgenic yeast strains was inhibited; however, growth of the *Δycf1* strain expressing *SaHsfA4c* was superior to that of the *Δycf1* strain transfected with the EV (**Figure 3A**). In liquid media supplemented with 15 μM CdCl_2_, the growth of yeast cells expressing *SaHsfA4c* was higher than that of the EV and *InSaHsfA4c* (**Figure 3B**). Furthermore, the Cd content of the strain overexpressing *SaHsfA4c* was significantly higher than that of the other two strains. The Cd contents of yeast overexpressing *SaHsfA4c* and *InSaHsfA4c* were respectively 2.8- and 0.24-fold higher than that of the EV-transfected strain (**Figure 3C**). These results indicated that the yeast expressing *SaHsfA4c* showed increased Cd uptake and accumulation, and that growth of transgenic yeast was promoted by *SaHsfA4c* under Cd stress.

Comparison of the nucleotide sequences of the two transcripts (*SaHsfA4c* and *InSaHsfA4c*) revealed the presence of an intron between exons 1 and exons 2 of *InSaHsfA4c* (**Figure 3D**). Heatster predicted the DBD of SaHsfA4c to contain a complete exon 1 and partial exon 2, whereas InSaHsfA4c was predicted to harbor a premature termination codon in the intron and contain an incomplete DBD domain (**Figures 3D, E**). These results suggested that the DBD of SaHsfA4c is critical for Cd resistance.

### *SaHsfA4c* Enhances Cd Tolerance in Transgenic *Arabidopsis* and Rescues Cd-Sensitive Defects of the *athsfa4c* Mutant

**Figure 4:**
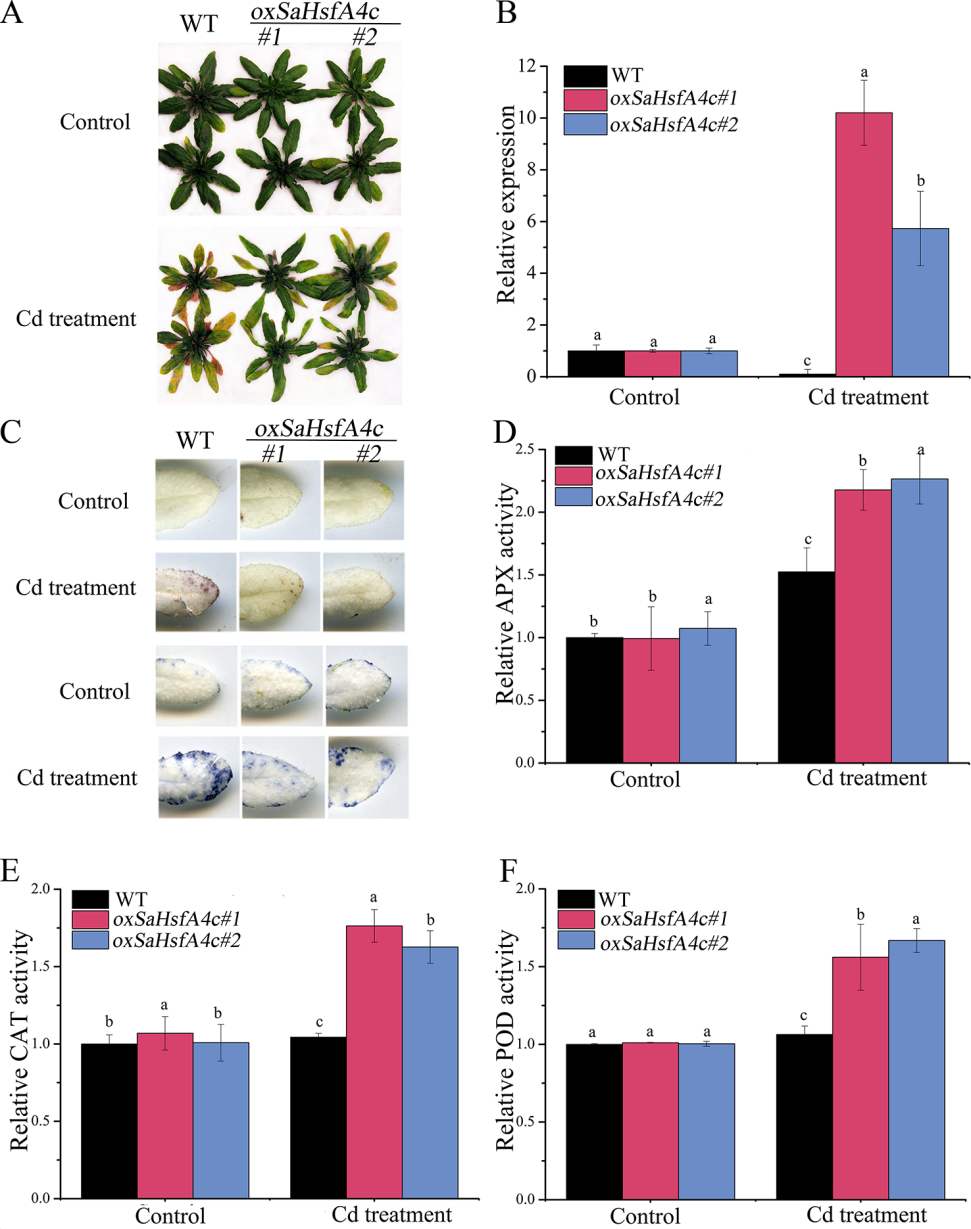
*SaHsfA4c* exhibits resistance to CdCl_2_ stress in transgenic *Arabidopsis*. **(A)** Growth status. **(B)** Relative expression level of *SaHsfA4c* in transgenic *Arabidopsis*. **(C)** DAB staining (upper) and NBT staining (lower). **(D)** Relative APX activity in the leaf. **(E)** Relative CAT activity in the leaf. **(F)** Relative POD activity in the leaf. Control, without Cd treatment; Cd treatment, 30 µM CdCl_2_ treatment for one week. Different letters on the bars indicate significant difference between WT and other lines. Bars indicate means ± standard deviations (SDs) of at least three independent biological experiments. Different letters on the bars indicate significant difference among multiple groups according to Tukey’s multiple range test (*p* = 0.05).

Ten transgenic *Arabidopsis* lines were obtained, among which two lines (*oxSaHsfA4c*#*1* and *oxSaHsfA4c*#*2*) expressing high levels of *SaHsfA4c* were selected for subsequent experiments (**Supplementary Figure S1A**). Higher expression levels of *SaHsfA4c* were observed in *oxSaHsfA4c*#*1* and *oxSaHsfA4c*#*2* under the non-stress condition (**Supplementary Figure S1B**). After Cd treatment for 7 days, the uppermost leaves of *oxSaHsfA4c*#*1* and *oxSaHsfA4c*#*2* plants remained green with only the basal leaves appearing slightly yellow, whereas most leaves of WT plants became severely wilted (**Figure 4A**). In addition, Cd stress up-regulated *SaHsfA4c* expression in the transgenic lines *oxSaHsfA4c*#*1* and *oxSaHsfA4c*#*2* (**Figure 4B**).

**Figure 5:**
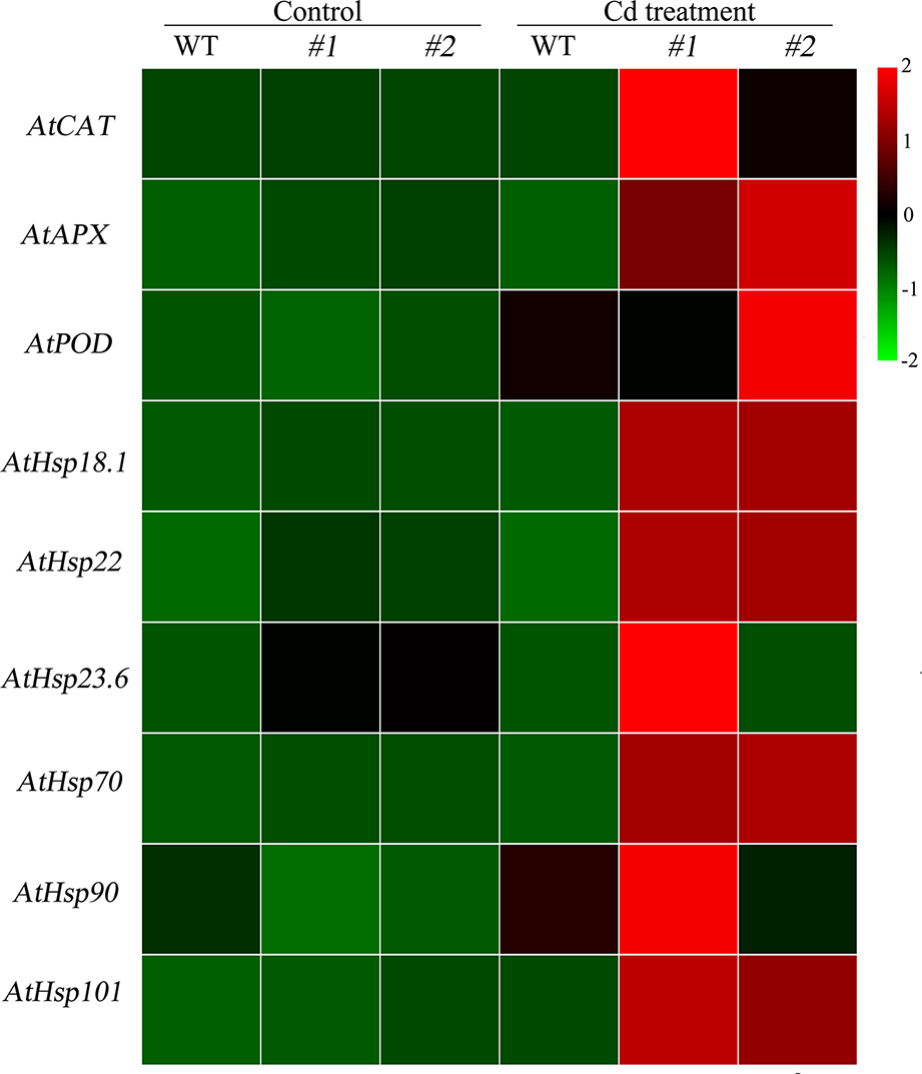
Expression patterns of ROS related genes and *Hsps* in transgenic *Arabidopsis* expressing *SaHsfA4c*. *#1* and *#2* represent the transgenic lines *oxSaHsfA4c#1* and *oxSaHsfA4c#2*, respectively. Control, without Cd treatment; Cd treatment, 30 µM CdCl_2_ treatment for one week. The red and green colors were used to represent high and low expression values, respectively.

Physiological responses to Cd stress were compared between the WT and the two transgenic *Arabidopsis* lines (*oxSaHsfA4c*#*1* and *oxSaHsfA4c*#*2*). Histochemical staining revealed that significantly less H_2_O_2_ and O_2_^−^ accumulated in the transgenic *Arabidopsis* lines (*oxSaHsfA4c*#*1* and *oxSaHsfA4c*#*2*) than in the WT under Cd treatment (**Figure 4C**). The activities of ROS-scavenging enzymes in the WT and transgenic lines were assayed to elucidate the role of *SaHsfA4c* in ROS homeostasis. Under the non-stress condition, APX, CAT, and POD activities were slightly higher in the transgenic *Arabidopsis* lines than those in the WT. Furthermore, the transgenic plants showed 1.2- to 3.0-fold higher APX, CAT and POD activities than the WT plants after Cd treatment (**Figures 4D–F**, **Supplementary Figure S2**). Next, the expression levels of ROS homeostasis-related genes (encoding ROS -scavenging enzymes), including *AtAPX*, *AtCAT* and *AtPOD*, and the downstream target genes of *Hsfs*, *AtHsp18.1*, *AtHsp22, AtHsp23.6*, *AtHsp70, AtHsp90* and *AtHsp101*, were analyzed by qRT-PCR. Although, ROS scavenging related genes in leaves of all tested lines were significantly up-regulated after Cd treatment, the expression levels of *oxSaHsfA4c*#*1* and *oxSaHsfA4c*#*2* lines were markedly higher than those of the WT (**Figure 5**).

**Figure 6:**
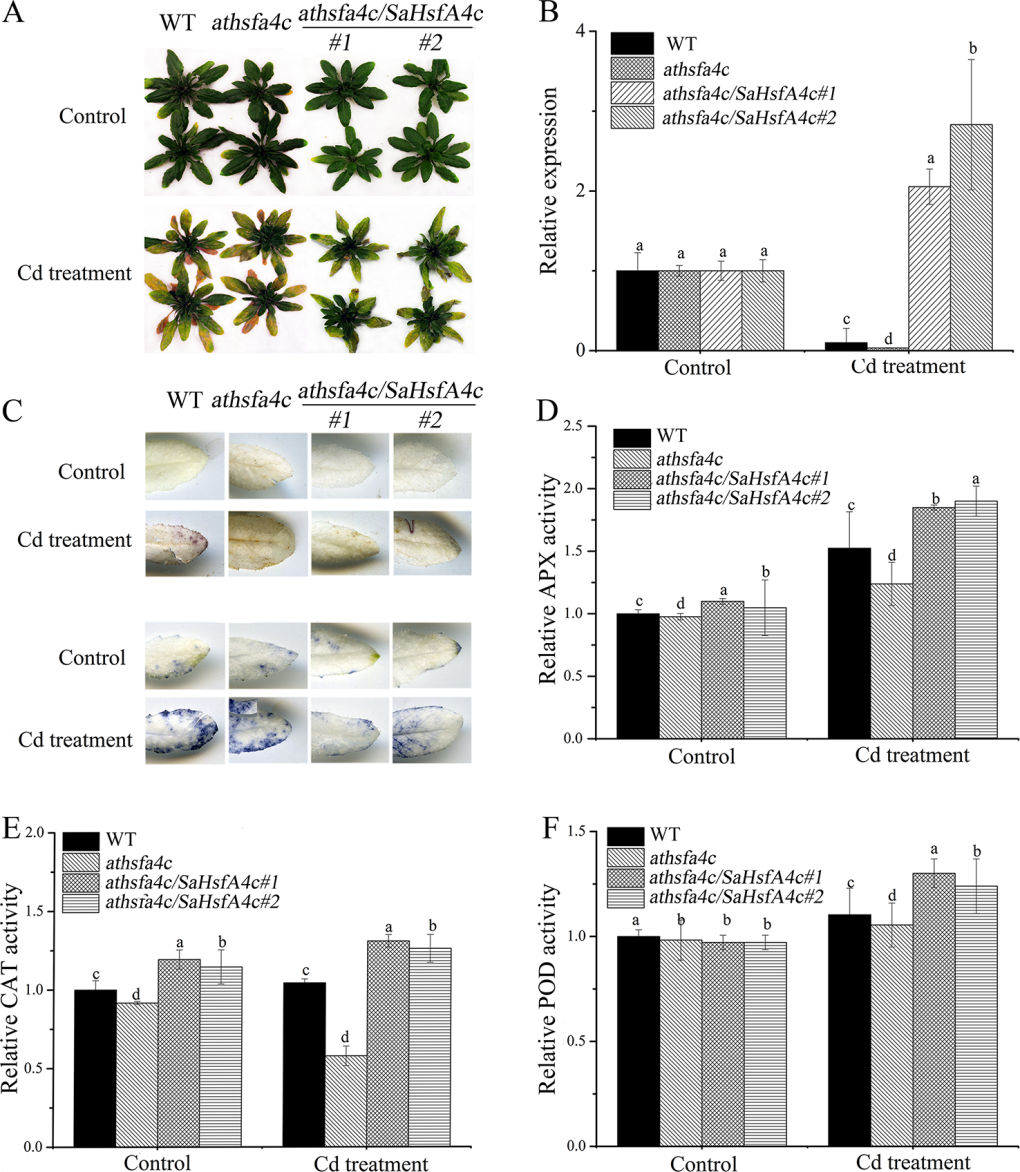
Characterization of *Arabidopsis athsfa4c* mutant transforming *SaHsfA4c*. **(A)** Growth status. **(B)** Relative expression level of *SaHsfA4c*. **(C)** DAB staining (upper) and NBT staining (lower). **(D)** Relative APX activity in the leaf. **(E)** Relative CAT activity in the leaf. **(F)** Relative POD activity in the leaf. Control, without Cd treatment; Cd treatment, 30 µM CdCl_2_ treatment for one week. Bars indicate means ± standard deviations (SDs) of at least three independent biological experiments. Different letters on the bars indicate significant difference among multiple groups according to Tukey’s multiple range test (*p* = 0.05).

To further elucidate the functions of *SaHsfA4c* in regulating Cd tolerance, the plant expression vector harboring *SaHsfA4c* was also introduced into the Cd-stress-sensitive *Arabidopsis athsfa4c* mutant. Two transgenic lines (*athsfa4c/SaHsfA4c*#*1* and *athsfa4c/SaHsfA4c*#*2*) showed significantly higher *SaHsfA4c* expression levels than the WT and *athsfa4c* mutant (**Supplementary Figure S1C**). Under the control condition, growth status was not significantly different among the four lines (WT, *athsfa4c*, *athsfa4c/SaHsfA4c*#*1* and *athsfa4c/SaHsfA4c*#*2*). After Cd treatment for 7 days, both transgenic lines exhibited less yellowing compared with that of the WT (**Figure 6A**). In addition, *SaHsfA4c* was up-regulated in the transgenic lines (*athsfa4c/SaHsfA4c*#*1* and *athsfa4c/SaHsfA4c*#*2*) by Cd treatment, with its expression increasing by 2.05- and 2.83-fold compared with that of the control (**Figure 6B**).

Histochemical staining revealed that markedly less H_2_O_2_ and O_2_^−^ accumulated in rescue *Arabidopsis* lines (*athsfa4c/SaHsfA4c*#*1* and *athsfa4c/SaHsfA4c*#*2*) under Cd treatment, whereas ROS accumulation was detected in the leaves of both the WT and the mutant line (**Figure 6C**). We also measured the activities of ROS-scavenging enzyme activities. Even under the non-stress condition, APX activity in the root and CAT activity level in the leaf of rescued plants were 1.15- to 1.3-fold higher than those of WT plants, which suggested that the increased *SaHsfA4c* expression of rescued plants resulted in higher ROS-scavenging enzyme activities. Furthermore, the rescue lines showed 1.25- to 2.2-fold higher APX and CAT activities than those of WT plants after Cd treatment (**Figures 6D, E**, **Supplementary Figure S3**). Activities of POD in the transgenic *Arabidopsis* lines were not significantly different from those of the WT under the non-stress condition but were 1.2- to 1.3-fold higher than those in the WT after Cd treatment (**Figure 6F**, **Supplementary Figure S3**). In summary, APX, CAT, and POD activities of *athsfa4c/SaHsfA4c*#*1* and *athsfa4c/SaHsfA4c*#*2* markedly increased in both the leaf and root under Cd treatment, and were higher than those detected in the WT and *athsfa4c* mutant.

### Overexpression of *SaHsfA4c* Confers Cd Tolerance in NHE *S. alfredii*

**Figure 7:**
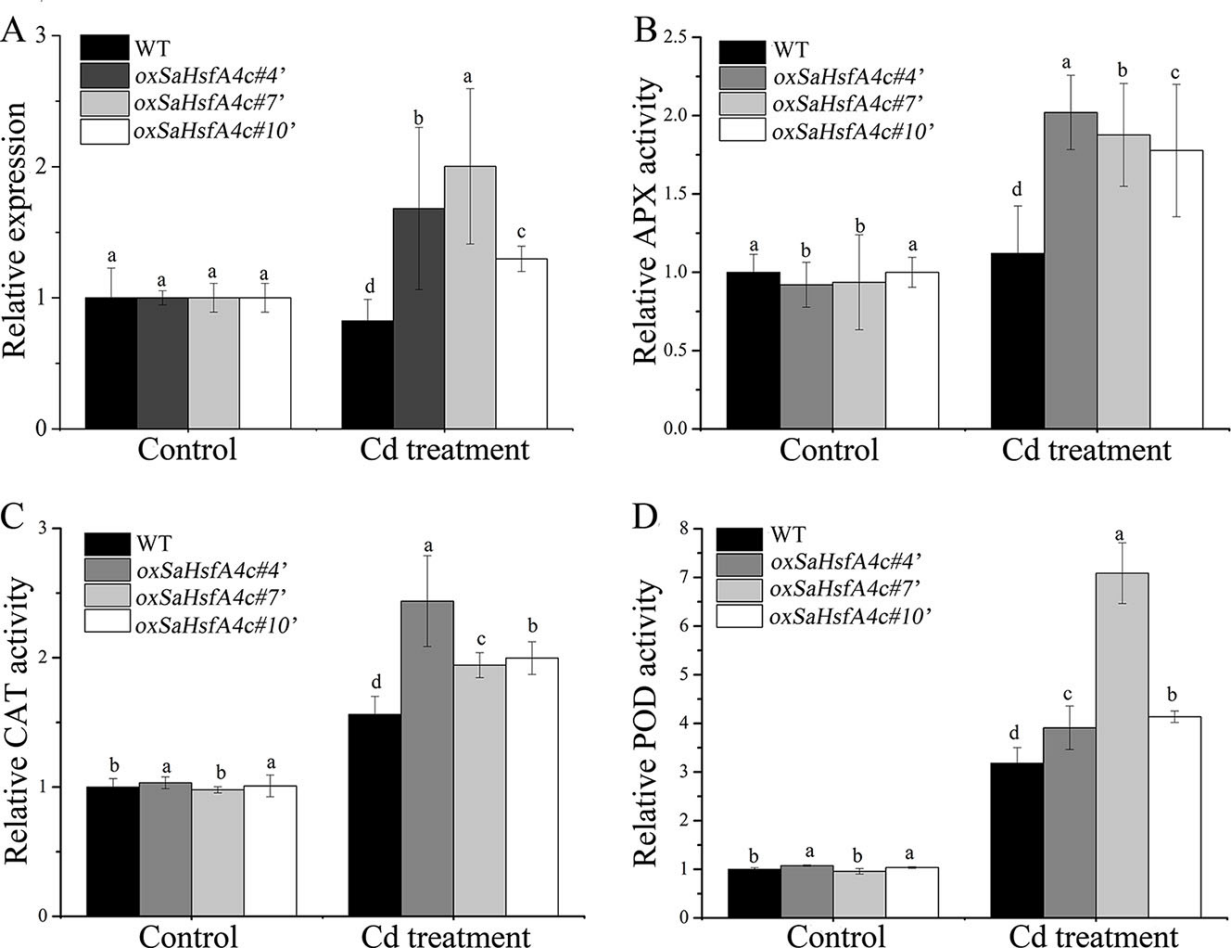
*SaHsfA4c* exhibits resistance to CdCl_2_ stress in transgenic NHE *S. alfredii* lines. **(A)** Relative expression level. **(B)** Relative APX activity in the leaf. **(C)** Relative CAT activity in the leaf. **(D)** Relative POD activity in the leaf. Control, without Cd treatment; Cd treatment, 400 µM CdCl_2_ treatment for one week. Bars indicate means ± standard deviations (SDs) of at least three independent biological experiments. Different letters on the bars indicate significant difference among multiple groups according to Tukey’s multiple range test (*p* = 0.05).

After validating their higher expression levels of *SaHsfA4c* under the control condition (**Supplementary Figure S1D**), three independent *SaHsfA4c*-overexpressing transgenic NHE *S. alfredii* lines (*oxSaHsfA4c*#*4′*, *oxSaHsfA4c#7′* and *oxSaHsfA4c#10′*) were selected for further study. After 400 µM CdCl_2_ treatment, *SaHsfA4c* expression was significantly increased in these three transgenic lines but was slightly reduced in the WT (**Figure 7A**).

Plant physiological responses to Cd stress were also analyzed. As in *Arabidopsis*, activities of the ROS-scavenging enzymes APX, CAT, and POD in the leaf and root of the transgenic lines were higher than those of the WT, both under the control condition and under Cd stress (**Figures 7B–D**, **Supplementary Figure S4**).

**Figure 8:**
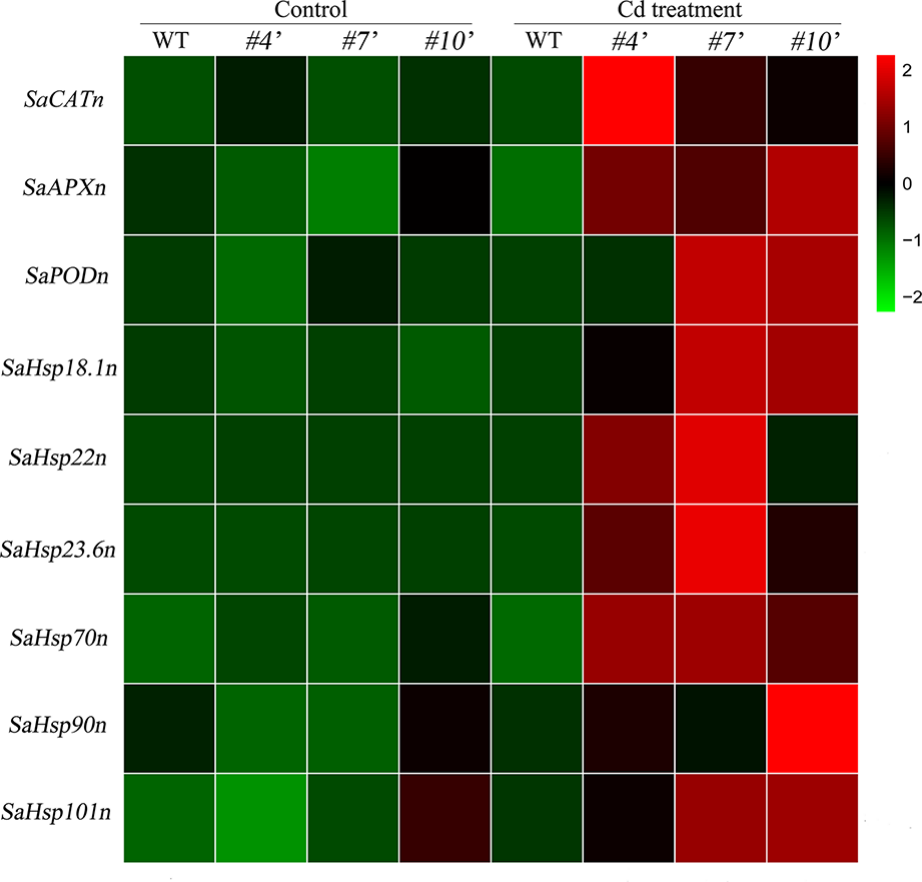
Expression patterns of candidate genes were performed in transgenic NHE *S. alfredii* overexpressing *SaHsfA4c*. *#4′*, *#7′* and *#10′* represent the transgenic lines *oxSaHsfA4c*#*4′*, *oxSaHsfA4c#7′* and *oxSaHsfA4c#10′*, respectively. Control, without Cd treatment; Cd treatment, 400 µM CdCl_2_ treatment for one week. The red and green colors were used to represent high and low expression values, respectively.

To evaluate the regulatory roles of *SaHsfA4c* in NHE *S. alfredii*, the homologs of *Arabidopsis’* ROS-scavenging related genes and *Hsps* were examined. All of these genes were up-regulated under Cd stress. Transcript levels of the tested genes were significantly higher in *oxSaHsfA4c*#*4′*, *oxSaHsfA4c#7′* and *oxSaHsfA4c#10′* than in the WT under Cd stress (**Figure 8**).

### Co-Expression Network of *SaHsfA4c* in HE *S. alfredii*

**Figure 9:**
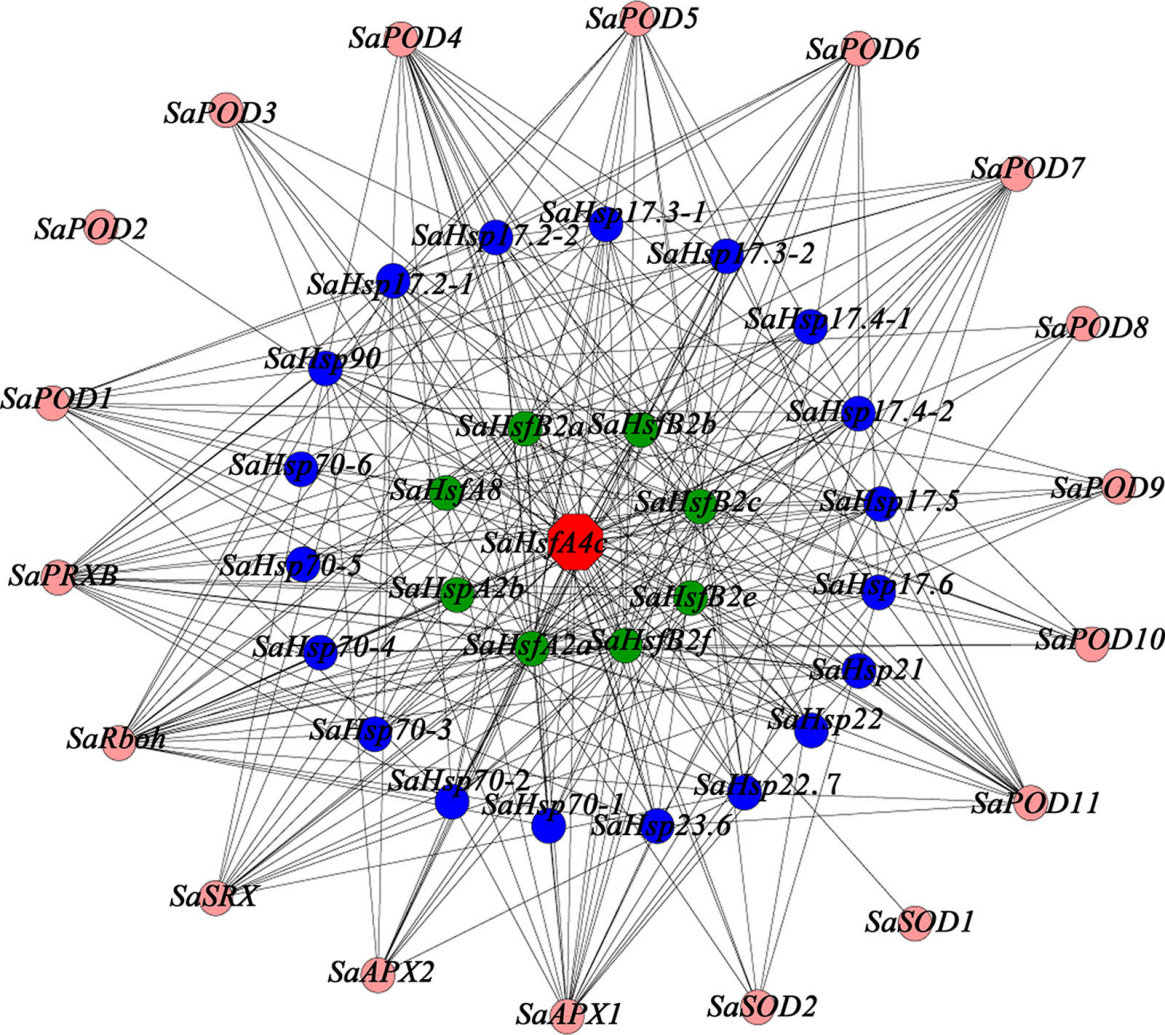
Co-expression network for *SaHsfA4c*. The red node is *SaHsfA4c*, pink nodes are genes related to encode ROS-scavenging enzymes, blue nodes are *Hsps* and green nodes are other *Hsfs*.

To provide additional context for the proposed function of *SaHsfA4c*, a gene co-expression analysis and GO functional classification were conducted using the transcriptome database obtained from HE *S. alfredii* under Cd stress (Han et al., 2016; **Supplementary Figure S5**). On the basis of the above mentioned results, which suggested that *SaHsfA4c* enhanced Cd tolerance by regulating ROS homeostasis and *Hsp*’ expression, we searched for relevant genes in the transcriptome data. A total of 45 genes were identified in the co-expression network (**Figure 9**). Notably, 18 genes in the co-expression network have been shown to be associated with ROS: 11 *PODs*, two *APXs* and two *SODs*. In addition, 19 *Hsps* with different molecular weight and eight *Hsfs*, which mainly belonged to the A and B subclasses, were identified as co-expressed genes.

## Discussion

In this study, *SaHsfA4c*, an ortholog of *Hsf*, was cloned from HE *S. alfredii*, and the functional properties and potential role of the gene in yeast and plants were characterized. The results showed that *SaHsfA4c* plays a role in Cd tolerance. It is proposed that this tolerance is mediated by target genes (ROS-related genes and *Hsps*) on the basis of two observations. First, *SaHsfA4c* was up-regulated under Cd stress, with higher transcript levels detected in the root, stem, and leaf (**Figure 2**). Second, ROS-related genes and *Hsps* were up-regulated in transgenic plants overexpressing *SaHsfA4c* (**Figures 5** and **8**).

In a previous study, *TaHsfA4a* and *OsHsfA4a* transgenic yeast exhibited Cd tolerance, and the growth status of the yeast strain overexpressing *TaHsfA4a* was superior to that of the control in liquid culture with CdCl_2_ (Shim et al., 2009). Similarly, the heterologous expression of *SaHsfA4c* improved Cd tolerance and accumulation in yeast in the current study. Compared with the control (EV), Δ*ycf1* harboring a fusion vector (pYES-DEST52-*SaHsfA4c*) showed enhanced growth (**Figure 3A**), whereas *InSaHsfA4c* did not. Our detailed analyses of the functional domains of the two different transcripts of *SaHsfA4c* revealed that *SaHsfA4c* containing the completed DBD enhanced Cd resistance in yeast; in contrast, *InSaHsfA4c* with a truncated DBD did not improved Cd resistance, which indicated that the DBD is critical for Cd tolerance (**Figure 3**). We therefore speculate that the DBD activates or enhances other factors, thereby promoting the expression of target genes. These results suggested that *SaHsfA4c* can compensate for the Cd sensitivity of yeast mutants, and that the DBD is essential for Cd resistance and accumulation.

Reactive oxygen species, which are induced by abiotic stresses, are harmful to plant growth and development (Li et al., 2017). In the *athsfa4a* mutant, the H_2_O_2_ content is much higher than that of the WT, and the accumulation of H_2_O_2_ in *atHsfA4a-*overexpressing plants is lower than that in the WT under salinity stress (Guo et al., 2016). Plant response to stress-induced ROS production is important to protect the stability of intracellular ROS levels (Azarabadi et al., 2017). To protect plant cells from oxidative damage, plants use a high effective enzymatic antioxidant defense system involving ROS scavenging enzymes, such as APX, POD and CAT (Gill and Tuteja, 2010). Many studies have revealed crosstalk between *Hsfs*, ROS and ROS-scavenging enzymes. In *Populus ussuriensis*, *PuHSFA4a* can increase glutathione *S*-transferase activity to reduce ROS production and improve Zn resistance of roots by directly regulating the target gene *PuGSTU17* (Zhang et al., 2019). Transgenic *Arabidopsis* overexpressing maize *ZmHsf06* can enhance salt tolerance by increases POD and SOD activities (Li et al., 2015). *AtHsfA2* regulated the ability of *Arabidopsis* to cope with heat and oxidative stress by inducing *APX* expression (Li et al., 2005). Transgenic tobacco overexpressing *PeHsf* produced more CAT under salt stress (Shen et al., 2013). Activities of SOD, APX and CAT in transgenic chrysanthemum overexpressing *CmHsfA4*, were higher than those of WT lines under salt stress (Li et al., 2017). In addition, *Hsf* activates the expression of downstream genes (including *Hsps*) and shows a similar pattern of expression to protect plants from external stress (Snyman and Cronjé, 2008; Xue et al., 2014; Xue et al., 2015; Chen et al., 2018). In the present study, we also observed that transgenic *Arabidopsis* and NHE *S. alfredii* expressing *SaHsfA4c* showed higher CAT, POD, and APX activities than those of the WT after Cd stress, and were able to induce the expression of ROS-scavenging system related genes (*POD*, *CAT* and *APX*) and *Hsps*. These results indicate that *SaHsfA4c* enhances the Cd tolerance of transgenic plants by activating ROS-scavenging enzyme activities and up-regulating *Hsps*.

Heavy metal tolerance and accumulation in plants is associated with a highly complex regulatory network system involving multiple genes. This system includes genes that are involved in diverse functions, such as metal ion absorption and transport, sequestration, chelation, detoxification and signal transduction (Luo et al., 2016). In the present study, the expression level of *SaHsfA4c* was high in the stem under the non-stress condition, and the expression levels in all tested tissues were also increased after Cd treatment. Although we observed that ROS-scavenging enzyme-related genes and *Hsps* may be the downstream target genes of *SaHsfs*, these genes are not the only target of *SaHsfs* in the regulation of Cd tolerance, which is similar to the finding that overexpression of *CUP1* in yeast does not satisfy the Cd tolerance of *TaHsfA4a* (Shim et al., 2009). A co-expression subnetwork with *SaHsfA4c* as a hub gene was also constructed using a transcriptome database of HE *S. alfredii* under Cd stress, from which 45 genes, including *Hsfs*, *Hsps* and ROS scavenging genes, were detected (**Figure 9**). Multiple major pathways and numerous target genes may participate in the Cd tolerance mechanism induced by *SaHsfA4c*. Further identification of the target genes of *SaHsfA4c* may provide new insights into the mechanism of Cd tolerance.

## Data Availability Statement

All datasets for this study are included in the article/**Supplementary Material**.

## Author Contributions

SC, XH, and RZ planned and designed the research. SC performed the experiments. MY, HL, YW, and ZL helped the plant culture and assisted some experiments. LW and YZ contributed analytical tools, LW contributed the design of the work. ML and GQ helped to modify the manuscript, and SC, XH and RZ wrote the manuscript and coordinated its revision. RZ contributed reagents/materials/funds support. MY helped to proof the final version. All authors read and provided helpful discussions, and approved the final version.

## Funding

This work was supported by the National Natural Science Foundation of China (No. 31872168); the National Nonprofit Institute Research Grant of Chinese Academy of Forestry (CAFYBB2017ZY007 and CAFYBB2016SY008); and the National Key R&D Program of China (No. 2016YFD0800801).

## Conflict of Interest

The authors declare that the research was conducted in the absence of any commercial or financial relationships that could be construed as a potential conflict of interest.
