# Supplementary material for: SaHsfA4c From Sedum alfredii Hance Enhances Cadmium Tolerance by Regulating ROS-Scavenger Activities and Heat Shock Proteins Expression
Source: Front Plant Sci. 2020 Feb 28;11:142. doi: 10.3389/fpls.2020.00142 (PMC7058639; doi:10.3389/fpls.2020.00142)
Supplement: Supplementary file 1 [file DataSheet_1.pdf]

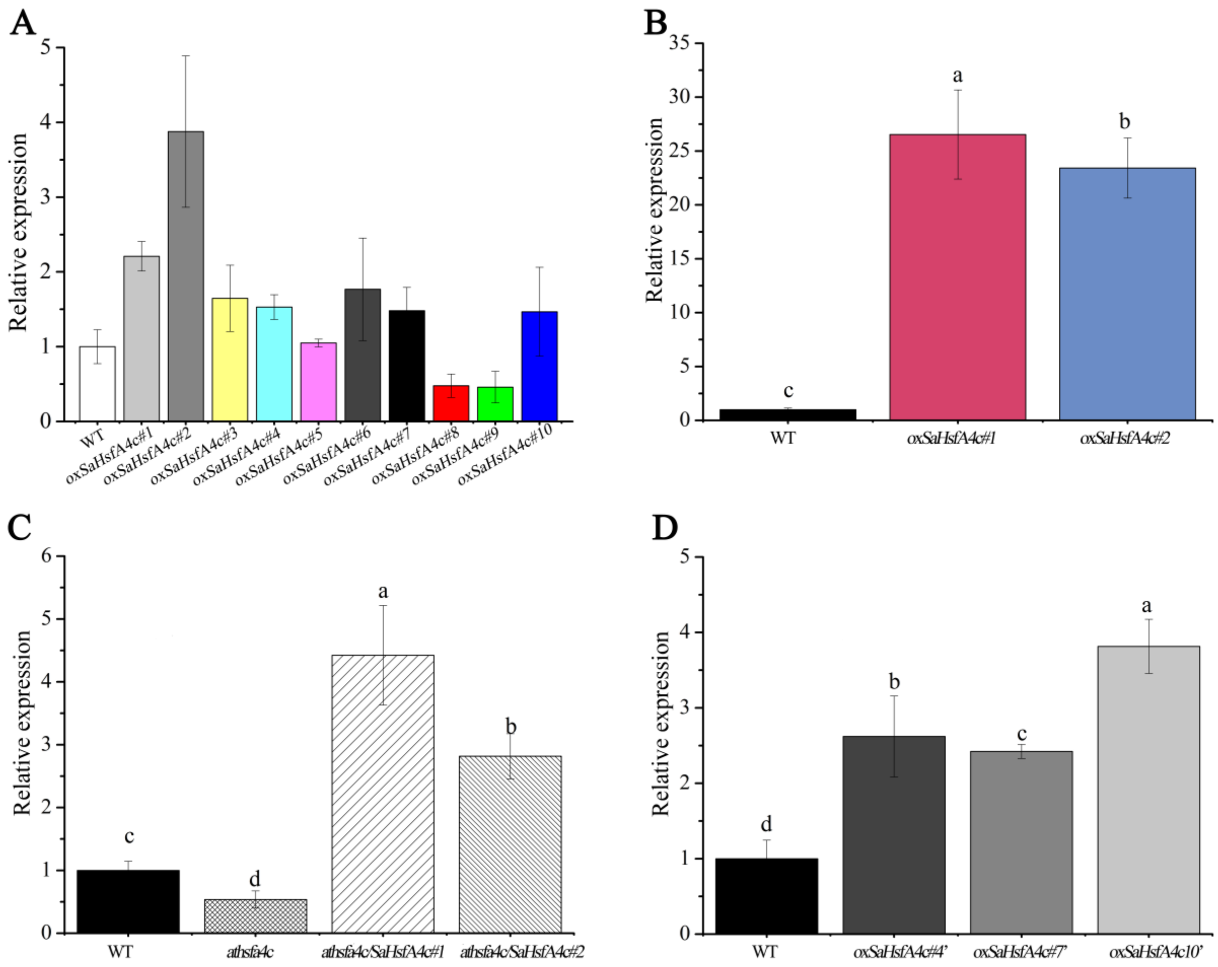

Figure S1. Relative expression levels of *SaHsfA4c* under normal condition. **(A)** qRT-PCR detection of *SaHsfA4c* expression in 11 transgenic *Arabidopsis* lines. **(B)** Relative expression of *SaHsfA4c* in transgenic *Arabidopsis* lines which selected for further experiments. **(C)** Relative expression of *SaHsfA4c* in *Arabidopsis* mutant and rescue lines. **(D)** Expression levels of *SaHsfA4c* in transgenic NHE *S. alfredii* lines which selected for further experiments.

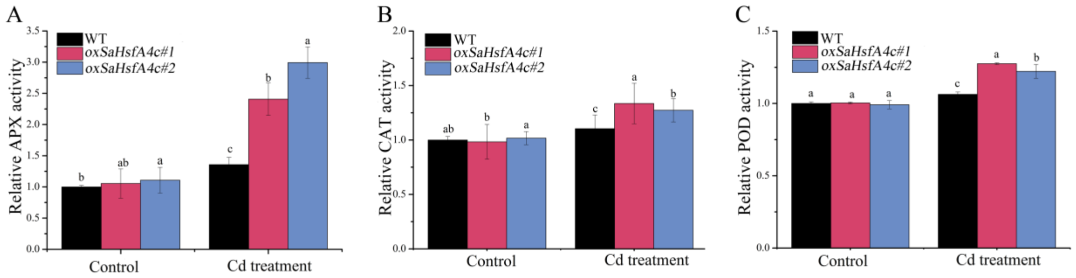

Figure S2. Relative ROS scavenger activities of WT and transgenic *Arabidopsis* lines overexpressing *SaHsfA4c* in the root. **(A)** Relative APX activity. **(B)** Relative CAT activity. **(C)** Relative POD activity. Control, without Cd treatment; Cd treatment, 400 μM $CdCl_2$ treatment for one week.

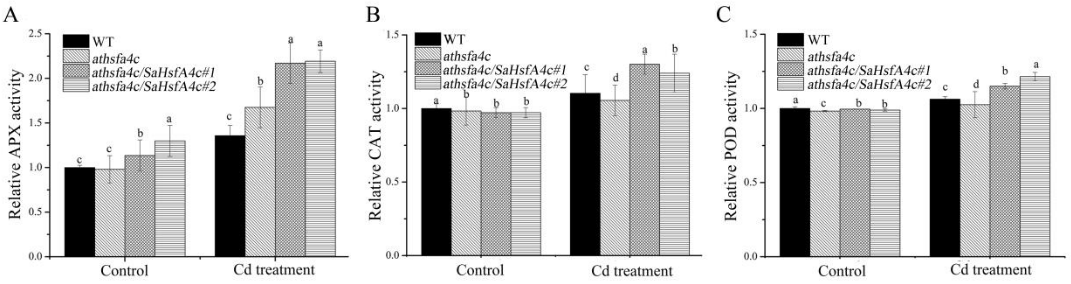

Figure S3. Relative ROS scavenger activities of WT and complementation mutant lines of *Arabidopsis* in the root. **(A)** Relative APX activity. **(B)** Relative CAT activity. **(C)** Relative POD activity. Control, without Cd treatment; Cd treatment, 400 μM $CdCl_2$ treatment for one week.

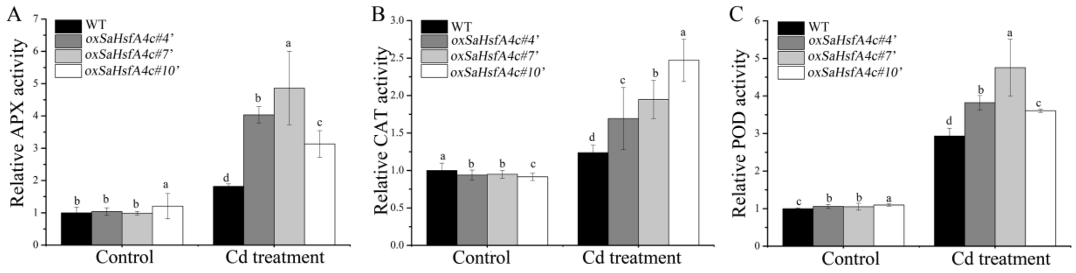

Figure S4. Relative ROS scavenger activities of WT and transgenic NHE lines overexpressing *SaHsfA4c* in the root. **(A)** Relative APX activity. **(B)** Relative CAT activity. **(C)** Relative POD activity. Control, without Cd treatment; Cd treatment, 400 μM $CdCl_2$ treatment for one week.

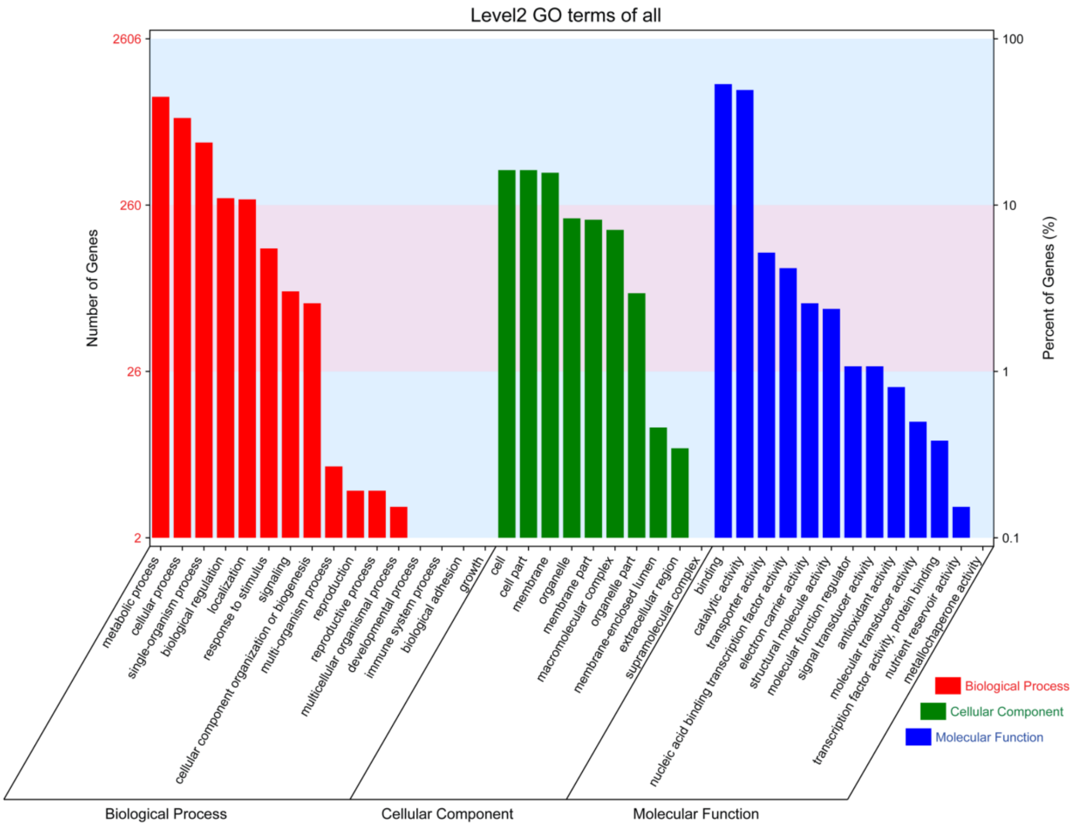

Figure S5. Number of genes assigned to each category by osgo online tool analysis.
